# Editorial: Current trends and future management of IBD, volume II

**DOI:** 10.3389/fmed.2025.1637213

**Published:** 2025-06-16

**Authors:** Sérgio Bronze, Iago Rodríguez-Lago, Triana Lobatón, Maria Manuela Estevinho

**Affiliations:** ^1^Department of Gastroenterology, Unidade Local de Saúde de Santa Maria, Lisboa, Portugal; ^2^Faculty of Medicine, University of Lisbon, Lisboa, Portugal; ^3^Gastroenterology Department, Hospital Universitario de Galdakao, Biocruces Bizkaia Health Research Institute, Galdakao, Spain; ^4^Department of Gastroenterology, Ghent University Hospital, Ghent, Belgium; ^5^Unit of Pharmacology and Therapeutics, Department of Biomedicine, Faculty of Medicine, University of Porto, Porto, Portugal; ^6^Department of Gastroenterology, Unidade Local de Saúde Gaia e Espinho, Vila Nova de Gaia, Portugal

**Keywords:** Crohn's disease, ulcerative colitis, inflammatory bowel disease, fatigue, extraintestinal manifestation

The management of inflammatory bowel disease (IBD), encompassing Crohn's disease (CD) and ulcerative colitis (UC), remains a global challenge due to its chronic, relapsing nature and substantial impact on patients' quality of life. Despite major therapeutic advances, important gaps persist in our understanding of disease biology, patient needs, and the long-term burden of illness. This second edition of *Current trends and future management of IBD, volume II* brings together a diverse collection of papers reflecting a growing shift toward more personalized, mechanistically informed, patient-centered care.

A central theme in this collection is the integration of mechanistic insights into clinical decision-making. Tian et al. interrogated the Blood Cell Consortium and the IEU Open GWAS project, where they applied Mendelian randomization to examine causal links between circulating leukocytes and IBD risk. Their analysis identified neutrophils as risk-enhancing and CD14^−^CD16^+^ monocytes as protective, reinforcing the pivotal role of innate immunity in disease pathogenesis. These genetics-based findings strengthen the rationale for biomarker development and pave the way for tailored therapeutic strategies.

While novel therapeutics have significantly advanced IBD management, a considerable proportion of patients continue to require the development of new drugs and alternative approaches due to the refractory course of the disease. This need is especially pronounced in underrepresented populations, such as pediatric and elderly patients, as the evidence in these subgroups is more limited. In this context, Kim et al. compared the infliximab originator with its biosimilar, CT-P13, in a pediatric population across multiple real-world outcomes. Their multicenter study demonstrated no significant differences in clinical remission, endoscopic healing, or drug persistence. These findings provide reassuring evidence supporting the use of biosimilars in children with IBD.

In addition to the importance of controlling the underlying inflammatory process, several studies in this collection highlight broader aspects of patient wellbeing. A growing consensus indicates the necessity of looking beyond biochemical and endoscopic remission. Patient-centered research is becoming increasingly important in this context. Schoefs et al. presented a solid protocol for a global discrete choice experiment to measure how patients prioritize treatment and disease-related attributes. Developed through a multi-step, stakeholder-informed process, the survey encompasses 14 attributes and is available in 15 languages. Besides evaluating trade-offs among efficacy, side effects, and routes of administration, the study also explores how preferences differ across patient subgroups, providing essential insights for aligning drug development, regulatory decisions, and reimbursement policies with the aims that genuinely matter to patients. This emphasis on patient experience extends to often-overlooked symptoms such as fatigue, disability, and psychosocial distress. Truyens et al. also provided a comprehensive review of IBD-related fatigue, proposing dysfunction of the gut–brain axis as a potential underlying mechanism. By reframing fatigue as both a secondary symptom and an independent clinical challenge, the authors underscore the need for targeted therapeutic approaches.

Similarly, Nardone et al. examined the psychosocial burden of IBD-related disability. Even in remission, patients frequently experience bowel urgency, sexual dysfunction, and impaired fertility—issues that remain underrecognized in routine care. The authors advocate for a paradigm shift: from treating inflammation alone to pursuing holistic remission, in which emotional, physical, and functional wellbeing are co-primary goals. Therefore, the information provided in this collection underscores the importance of integrated care models that include mental health and social support services.

Collectively, the papers from this second volume of *Current trends and future management of IBD, volume II* highlight the multidimensional nature of IBD and the corresponding need for equally multifaceted management strategies. The future of IBD care will not be shaped solely by new therapeutics, but by integrating mechanistic insights, real-world evidence, and—most importantly—the patient's voice. From basic research on neutrophils and monocytes to patient perceptions, social impact, and the mental health consequences of the disease, the range of perspectives represented here reflects the field's ongoing evolution toward more equitable, individualized, and holistic care.

